# The Application of Epicardium in Heart Failure Treatment: Opportunities and Challenges

**DOI:** 10.7150/ijms.118408

**Published:** 2025-09-03

**Authors:** Chenlei Zhou, Yaping Xu, Zhikun Guo

**Affiliations:** 1 Henan Key Laboratory of Medical Tissue Regeneration, Xinxiang Medical University, Henan, P.R. China; 2 Henan Key Laboratory of Cardiac Remodeling and Transplantation, Zhengzhou Seventh People's Hospital, Henan, P.R. China.

## Abstract

Heart failure remains one of the leading causes of morbidity and mortality worldwide. Conventional treatment strategies, while beneficial, face numerous limitations. Drug therapies may lead to resistance, while device-based treatments such as LVAD and ICD carry risks of infection, bleeding, device failure, and high costs. For end-stage heart failure, heart transplantation is further constrained by donor shortages and immune rejection. In contrast, cell-based therapies have emerged as a promising alternative. Recent studies have highlighted the critical role of the epicardium and epicardium-derived cells (EPDCs) in cardiac regeneration. These cells contribute to heart repair through multiple mechanisms, including direct cell therapy, the development of epicardium-based biomaterials, and integration with gene therapy approaches. This review outlines the anatomical structure and biological functions of the epicardium, explores the regenerative potential of the epicardium and EPDCs, and evaluates their application in heart failure treatment. Furthermore, it discusses the translational potential and current challenges associated with epicardial-based therapies, offering novel insights and strategies for heart failure management.

## Introduction

Heart failure (HF) is a common and heterogeneous clinical syndrome characterized by high morbidity and mortality. It significantly impairs patients' quality of life and contributes to a substantial global health burden. Although the incidence of HF has stabilized or declined in high-income countries in recent years, the global prevalence continues to rise due to population aging and the increasing prevalence of risk factors, with a concerning trend toward younger onset^1^. The pathogenesis of HF involves a variety of factors, including ventricular systolic or diastolic dysfunction, valvular abnormalities, and disturbances in cardiac rhythm or conduction^2^. Current therapeutic strategies for HF mainly include pharmacological interventions, lifestyle modifications, surgical procedures, and heart transplantation. However, these approaches demonstrate limited efficacy in heart failure with preserved ejection fraction (HFpEF) and are further challenged by issues such as drug resistance, adverse effects, and high treatment costs^3^.

Cardiac regeneration relies on the generation of functional cardiomyocytes and the formation of a vascular network to support these newly formed cells^4^. In recent years, the epicardium has garnered increasing attention as a critical tissue in cardiac regeneration. Rich in progenitor cells, the epicardium plays a pivotal role in signal transduction during heart repair^5^. A subset of EPDCs has been shown to differentiate into cardiac fibroblasts and vascular smooth muscle cells, thereby providing essential structural and functional support to the myocardium^6^. Consequently, the epicardium holds significant therapeutic promise for HF.

Nevertheless, despite growing interest in the role of the epicardium in HF treatment, several challenges remain. Key among these are the need to effectively activate epicardial cells to promote myocardial regeneration^6^, and to address the potential for fibrosis during epicardial repair processes^7^, ^8^. This review aims to explore the therapeutic potential of the epicardium in HF, analyze its role in myocardial repair, and discuss the challenges and future directions of epicardial-based therapies.

## Biological Functions and Regenerative Potential of the Epicardium

**Figure 1:**
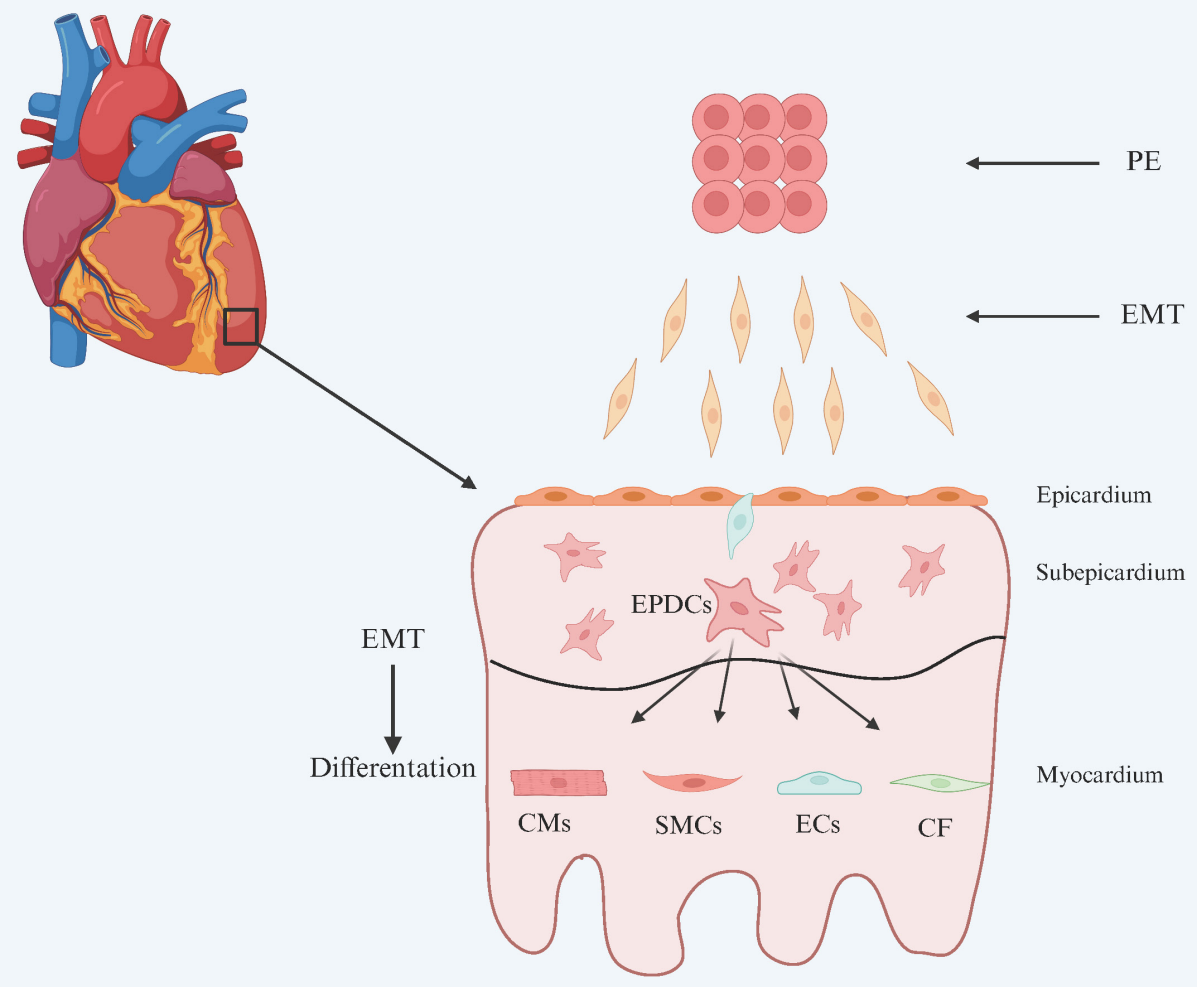
Development of the epicardium and its lineages. The epicardium originates from the proepicardium (PE). Proepicardial cells undergo epithelial-to-mesenchymal transition (EMT) and migrate to the myocardial surface of the looping heart, where they adhere, migrate, proliferate, and undergo mesenchymal-to-epithelial transition (MET) to form a squamous epithelial layer, the epicardium. While the epicardium remains an intact layer, some epithelial cells undergo a second round of EMT and migrate into the matrix-rich sub-epicardial layer. Fate mapping and genetic lineage tracing studies have demonstrated the multi-lineage potential of these EPDCs. EPDCs differentiate into smooth muscle cells (SMCs), contributing to the formation of coronary vasculature, and into cardiac fibroblasts (CF) in the mature heart. Although the contribution of EPDCs to cardiac endothelial cells (ECs) and cardiomyocytes (CMs) has been described, this remains a subject of ongoing debate. (The image has been adapted based on reference^12^.)

The pericardium is anatomically divided into the fibrous pericardium and the serous pericardium. The latter consists of a parietal layer and a visceral layer, with the visceral layer constituting the epicardium^9^. The epicardium is composed primarily of a single layer of flattened epithelial cells, which connect to the underlying myocardium through a layer of connective tissue^10^. During embryonic development, the epicardium serves two essential roles: it acts as a source of progenitor cells for various cardiac lineages, and it provides a paracrine environment conducive to cardiomyocyte compaction and maturation^11^. During development, a subset of epicardial cells undergoes epithelial-to-mesenchymal transition (EMT) and migrates into the myocardium, differentiating into various cell types that are essential for normal heart development (Figure 1). In addition, epicardial cells contribute to cardiac repair by generating multiple cell types and secreting paracrine factors.

Notably, the epicardium plays a central role in myocardial repair through multiple mechanisms. Its close interaction with the myocardium enables epicardial-derived signals to directly stimulate cardiomyocyte proliferation, while signals from cardiomyocytes induce EMT in epicardial cells, facilitating their differentiation into cardiomyocytes and coronary vascular cells^13^. Lavine *et al.* demonstrated that fibroblast growth factor (FGF) signaling between the epicardium and myocardium activates Hedgehog (Hh) signaling and induces vascular endothelial growth factor (VEGF) expression, thereby promoting coronary vessel formation^14^, ^15^. Huang *et al.* reported that suppression of CCAAT/enhancer-binding protein (C/EBP) signaling in the epicardium reduced fibrosis and improved contractile function post-myocardial infarction, likely by inhibiting neutrophil infiltration into the infarcted region^16^. Recent studies by Elizabeth *et al.* revealed that following ventricular amputation, neutrophils rapidly accumulate and secrete the epidermal growth factor ligand heparin-binding EGF-like growth factor (HBEGF), which acts in concert with other FGFs and MAPK/ERK pathways to induce epicardial regeneration and contribute to cardiac repair^17^. Additionally, Bargehr *et al.* found that exosomes derived from mouse epicardial cells contain microRNAs such as miR-30a, miR-100, and miR-27a, which promote the proliferation of human pluripotent stem cell-derived cardiomyocytes^18^.

The epicardium harbors a multipotent progenitor cell population that, upon undergoing EMT, loses cell-cell adhesion and apical-basal polarity while acquiring mesenchymal characteristics, enabling migration and invasion into the myocardium. These cells are known as EPDCs. EPDCs can differentiate into coronary vascular smooth muscle cells, cardiac fibroblasts, endothelial cells, and other cell types^19^-^21^. During EMT, EPDCs acquire mesenchymal properties and express mesenchymal proteins. Eroglu *et al.* demonstrated in a salamander cryoinjury model that CLDN⁺ epicardial cells formed honeycomb-like structures via tight junctions at the injury site, underwent transcriptional reprogramming, and ultimately differentiated into cardiomyocytes^22^.

However, in the adult heart, the epicardium remains quiescent and is only reactivated following cardiac injury. Compared to fetal development, the contribution of EPDCs to myocardial repair in adults is significantly reduced. Multiple studies have shown that most newly formed cells after injury originate from pre-existing cardiomyocyte subpopulations, rather than from newly differentiated EPDCs. Therefore, the role of epicardial cells in adult hearts is largely paracrine rather than serving as a direct source of reparative cells^16^, ^23^-^25^. EPDCs mostly remain on the heart surface and preferentially differentiate into cardiac fibroblasts and smooth muscle cells. They contribute to the survival and growth of coronary vessels after myocardial infarction through paracrine secretion of factors such as VEGFA and FGF2^26^-^28^. Moreover, unlike mesenchymal stem cells (MSCs), EPDCs uniquely express cardiac-specific marker genes such as GATA4 and cTnT. Comparative studies between primary human MSCs and EPDCs derived from the human right atrial appendage confirmed that only EPDCs exhibit these myocardial markers. Although EPDCs display osteogenic differentiation potential *in vitro* under conditions that promote MSCs differentiation into adipogenic and osteogenic lineages, they fail to differentiate into adipocytes or endothelial cells^29^.

Although EPDCs can reach injured sites and contribute to regeneration, their reparative capacity is often suppressed. As a result, EPDCs primarily exert their effects through the secretion of growth factors, cytokines, and extracellular matrix components that promote the proliferation, migration, and differentiation of resident cardiac cells. EPDCs stimulate the proliferation, migration and differentiation of resident cells by secreting growth factors, cytokines and extracellular matrix, thereby promoting tissue repair and regeneration. However, their regenerative potential is limited. Therefore, the epicardium is a powerful tool for new therapeutic strategies, which can synergistically repair damaged myocardium through differentiation into functional myocardium and endogenous response to paracrine stimulation^11^. EPDCs are known to secrete key factors such as retinoic acid and fibroblast growth factors, which are critical for cardiomyocyte proliferation. Moreover, GATA4 and GATA6 signaling within the epicardium regulates the development of the cardiac neural crest^30^-^32^. In zebrafish models, following acute cardiac injury, FGF signaling pathways are activated, with increased expression of FGFR2 and FGFR4 observed in EPDCs surrounding the atrium. FGFR4 remains highly expressed near the wound site even 30 days post-amputation. Inhibition of FGFR signaling in the epicardium disrupts the EMT process and neovascularization in the injured region^33^.

It is also noteworthy that the epicardium plays a crucial role in transport. During embryonic development, epicardial mesothelial cells actively transport fluids, cells, and particles across the serosa and pericardial cavity. Additionally, they synthesize and secrete mediators that regulate inflammation, immunity, and tissue repair^34^.

## Applications of the Epicardium in Heart Failure Therapy

**Figure 2:**
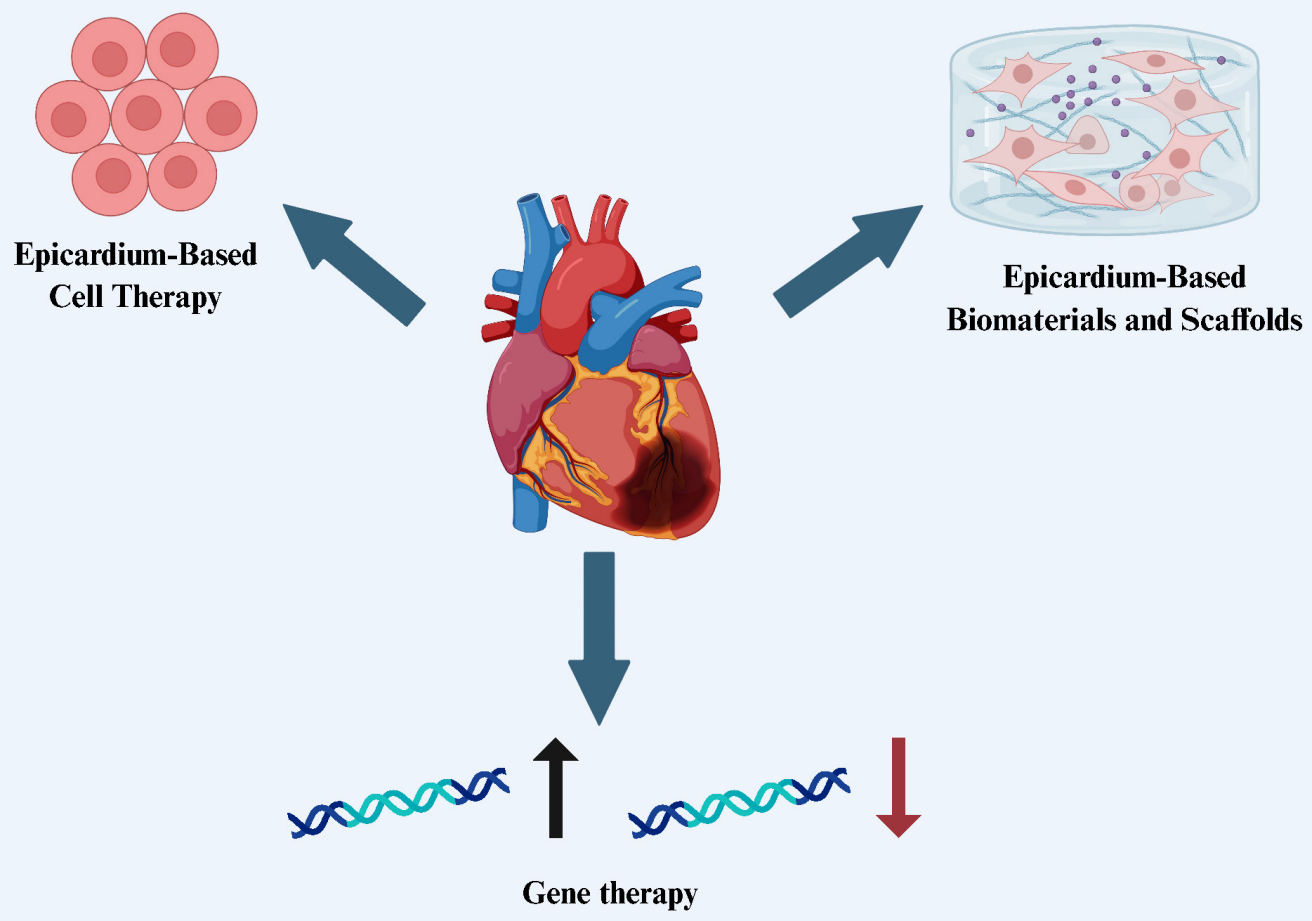
Therapeutic applications of the epicardium in heart failure

The epicardium possesses a range of critical biological functions and remarkable regenerative potential, making it a key focus in recent heart failure research. As our understanding of epicardial development and regenerative properties deepens, researchers are exploring their therapeutic applications in cardiac repair from multiple perspectives. Currently, the therapeutic potential of the epicardium is mainly reflected in three areas: (1) the use of epicardial and EPDCs for cell therapy, leveraging their multipotent differentiation capacity and paracrine effects to promote myocardial and vascular regeneration; (2) the development of epicardium-based biomaterials and tissue engineering scaffolds, providing mechanical support and a conducive microenvironment for cardiac repair; and (3) the use of the epicardium as a targeted delivery platform for gene therapy, aiming to enhance cardiac repair and functional recovery through the activation or editing of key signaling pathways (Figure 2). These strategies offer new avenues for precision therapy and clinical translation in the treatment of heart failure.

### Epicardium-Based Cell Therapy

EPDCs play a pivotal role in cardiac repair. Studies have demonstrated that EPDCs exhibit diverse differentiation potentials under different stimuli (Table 1). For instance, atrial natriuretic peptide can induce EPDCs differentiation into adipocytes, whereas angiotensin II promotes their differentiation into myofibroblasts^35^. Overexpression of Cdx1 enhances EMT in epicardial cells, facilitating their differentiation into coronary vascular smooth muscle cells (SMCs) and endothelial cells, while also promoting their migration into the myocardium^36^. Thymosin β4 (Tβ4) has been shown to enhance the migratory capacity of adult EPDCs, thereby augmenting their regenerative potential^37^. *In vitro* studies using chick and mouse epicardial explants have revealed that TGF-β receptor stimulation can induce EMT and differentiation into SMCs. Similarly, stimulation with platelet-derived growth factor (PDGF) receptors or FGF1/2/7 also promotes EMT and SMCs differentiation^5^. Dronkers *et al.* reported that EPDCs spontaneously undergo EMT *in vitro*; in adult EPDCs, this can be inhibited by the ALK5 kinase inhibitor SB431542, though its effect on fetal EPDCs is limited^38^. Furthermore, using an inducible large T-antigen (LT) expression system regulated by doxycycline, highly proliferative adult EPDCs can be obtained. Upon withdrawal of doxycycline, these cells retain epicardial morphology, EMT capacity, and paracrine signaling properties^39^.

In adult mammals, the heart has limited capacity to regenerate functional cardiomyocytes after injury, often resulting in inflammation and fibrotic scar formation. The epicardium and its derivatives are central to this fibrotic process. Typically quiescent in the adult heart, epicardial cells can be reactivated following myocardial injury by re-expressing progenitor markers such as Raldh2, Tbx18, and WT1. These activated cells undergo EMT and generate EPDCs, which subsequently differentiate into multiple cell types, including SMCs, endothelial cells (promoting neovascularization), and fibroblasts (facilitating collagen deposition)^36^, ^40^-^42^. Additionally, the epicardium contributes to cardiac repair by recruiting regulatory T cells to suppress excessive immune responses, limiting pathological remodeling, and by secreting paracrine factors that stimulate coronary vessel growth and improve myocardial regeneration^42^.

Nevertheless, the intrinsic response of EPDCs alone is insufficient for complete myocardial repair. For example, silencing intraflagellar transport protein 88 (IFT88), which is essential for ciliary function, promotes epicardial EMT and enhances Wnt/β-catenin signaling and bFGF expression. This leads to an increased number of WT1⁺ cells in both the epicardium and myocardium, facilitates EMT and angiogenesis after myocardial infarction, attenuates hypertrophic remodeling, and improves cardiac function^41^. Tcf21 is a critical target for regulating cardiac fibrosis. Loss of Tcf21 function in EPDCs enhances myofibroblast differentiation, while its overexpression suppresses this process. Lipid nanoparticles carrying lncRNA-TARID can upregulate Tcf21 expression in EPDCs, significantly improving cardiac function and histological outcomes in both mouse and porcine myocardial infarction models^40^. Oxytocin activates its receptor via the TGF-β signaling pathway and its downstream effectors, reprogramming epicardial cells toward a progenitor-like state^43^. Moreover, Sca1⁺ bone marrow cells from young mice can promote EPDCs proliferation, migration, and EMT reactivation via the TGF-β pathway, thereby enhancing cardiac regeneration^44^.

Epicardium is also a direct gateway for material exchange between pericardial fluid and myocardial tissue. The cells and molecular substances of pericardial fluid and myocardium can be freely interchanged through epicardium. The stem cells injected into the pericardial cavity can directly penetrate the epicardial mesothelial cells into the myocardial tissue and play a therapeutic role in heart failure^45^. The delivery of extracellular matrix, growth factors, drugs, bioactive materials and genetic materials through the pericardial cavity can smoothly reach the myocardium through the epicardium and play a role in the treatment of myocardial diseases. Transcardial mesothelial cell route not only overcomes the limitations of traditional systemic drug delivery methods, but also has a variety of intrinsic advantages compared with other methods, which can achieve greater therapeutic effect. Intrapericardial drug delivery shows versatility in dealing with some cardiac diseases. Continuous research in this field undoubtedly brings further innovation and progress hope for improving cardiac therapy^46^.

Collectively, these findings highlight the therapeutic potential of EPDCs in heart failure. By harnessing their multipotent differentiation capacity, EPDCs can promote myocardial regeneration, angiogenesis, and cardiac function recovery. Particularly, through the regulation of cell migration, EMT, and immune responses, EPDCs not only contribute to myocardial tissue repair but also enhance coronary vessel growth and mitigate pathological remodeling via paracrine signaling. These insights provide a promising foundation for the development of epicardium-based cell therapies in clinical heart failure treatment.

### Epicardium-Based Biomaterials and Scaffolds

Spindle-shaped epicardium-derived cells secrete various extracellular matrix (ECM) components, such as osteopontin and fibronectin^47^, ^48^. When combined with biomaterials, these cells can be used as injectable materials to enhance cell adhesion and reduce apoptosis. The epicardium also provides essential ECM components for vascular reconstruction and muscle regeneration^22^. Epicardial composite patches fabricated from human cardiac extracellular matrix (hcECM) can be processed into hydrogels (hgECM) and coated onto scaffolds. These constructs maintain nanoscale structural stability and mechanical properties while improving cell adhesion and reducing cell necrosis on the scaffold surface^49^. When mechanically integrated with the heart as structural components of the cardiac chamber, epicardial-mimicking patches exhibit elastic properties similar to those of the native epicardium. After eight weeks of implantation in a rat myocardial infarction model, these patches demonstrated electrical signal conduction, reduced wall stress, and improved contractile function^50^.

Reproducible activation of epicardial behavior has become a key therapeutic target in treating cardiac injury. Transitioning epicardial cells from an epithelial to a motile mesenchymal phenotype is crucial for their beneficial effects on the myocardium. The goal of engineered cardiac patches is to replicate the native ECM, provide mechanical support, and create a favorable environment for cell migration, integration, proliferation, and differentiation, while also facilitating efficient nutrient and waste exchange^51^. By suturing cardiac patches onto the epicardial surface, treatment areas can be precisely localized, enhancing therapeutic efficiency. When conditioned medium from epicardial cells is embedded into collagen nanofiber membranes and implanted into the heart, improvements in myocardial contractility and prevention of excessive fibrosis after infarction have been observed. Wei *et al.* found that the expression of follistatin-like 1 (Fstl1) in the epicardium decreases following myocardial infarction. Application of a human Fstl1 epicardial patch stimulated cell cycle re-entry and proliferation of existing cardiomyocytes, thereby improving cardiac function and survival in both mouse and pig models^52^.

Three-dimensional (3D) printing technology enables the precise construction of cardiac patches by depositing successive layers of biomaterials into a defined 3D structure. This technique allows for fine control over patch geometry, diameter, and pore size, promoting nutrient transport, enhancing cell proliferation, and ensuring adequate oxygen and nutrient supply within the scaffold^53^, ^54^. When designing cardiac patches, special attention must be paid to the anisotropic nature of the myocardium. In the longitudinal axis of the myocardium, intercalated discs and gap junction chains facilitate ionic current flow, while the transverse axis has fewer gap junctions, resulting in higher resistance. This anisotropy significantly affects myocardial conductivity. Improperly designed patches may exacerbate anisotropy and trigger arrhythmias^55^.

Given the contractile-relaxation cycle of cardiac activity, the elasticity of the patch is equally critical. Patch materials must exhibit excellent biocompatibility to support cell adhesion and growth, low immunogenicity, and a degradation rate matched to the pace of tissue remodeling. The patch's electrical conductivity should approximate that of native myocardium to ensure synchronized contraction and electrical pacing. Cardiac patches not only provide mechanical support to infarcted areas but also serve as a delivery platform for cells or bioactive factors, thereby improving cell retention and enhancing therapeutic efficacy. Although cell transplantation using patches has shown promise in myocardial repair, the number of surviving cells and their integration with infarcted tissue remain suboptimal. Furthermore, poor electromechanical coupling between the implanted patch and host myocardium may lead to arrhythmias. Nutrient diffusion also limits the thickness of patches, and currently, most designs are restricted to small animal models. Achieving clinically relevant, vascularized patch sizes remains a critical challenge that requires further investigation^56^.

### Integration of the Epicardium and Gene Therapy

Gene therapy involves the introduction of exogenous genes into cells *in vivo* or *in vitro* to achieve biological or therapeutic purposes. XC001, a novel adenovirus type-5 vector, is administered via a minimally invasive epicardial approach for the treatment of refractory angina. Volunteer trials have shown that XC001-VEGF gene therapy significantly improves both objective and subjective symptoms and has good tolerability^57^. However, research utilizing the epicardium as a gene delivery vector for gene therapy has yet to be fully explored^58^.

Epicardial gene therapy involves brushing a solution containing a polymer mixture, vector, and diluted protease onto the atrial epicardium. The protease facilitates vector penetration, thereby enabling widespread atrial delivery while minimizing off-target effects^59^. Studies have demonstrated that this approach can deliver high-density adenoviral (Ad) vectors to atrial myocardium in pigs without evidence of off-target expression in other organs^60^-^62^. This method circumvents blood and anatomical barriers, allowing the vector to reach all parts of the atrium via the epicardium. Clinically, this delivery strategy can be employed during open-heart surgery or heart transplantation and, after modifications, could also be applied using thoracoscopy or catheter-based techniques^63^. The SmartSleeve enhances the effectiveness of therapy by allowing suture-free, conformal adhesion to the epicardial surface and enabling controlled, on-demand drug delivery in response to electrocardiographic signals^64^.

The CRISPR/Cas9 system, which uses a 20-nucleotide guide RNA, has emerged as a powerful gene-editing tool with the potential to correct genetic mutations^65^, ^66^. By using epicardial magnets for site-specific application, the CRISPR/Cas9 system can be locally delivered to the heart, reducing off-target effects in other tissues and cells, and controlling the duration of gene editing at specific *in vivo* sites to maximize therapeutic efficacy. In non-human primate models, the development of stronger externally actuated CRISPR/Cas9 magnetic complexes and further advancement of CRISPR/Cas9-mediated targeted tissue therapeutic gene editing techniques will offer new avenues for cardiovascular disease treatment^67^. Wang *et al.* combined a functionalized self-assembling peptide (SAP) RADA16-I (RADA-RPR) with Tβ4, enabling its adhesion to EPDCs. The functionalized SAP-released Tβ4 effectively activated the epicardium, induced EPDCs differentiation into cardiovascular and lymphatic endothelial cells, and promoted cardiomyocyte proliferation. After four weeks of treatment in a mouse myocardial infarction model, adverse cardiac remodeling was significantly attenuated and cardiac function was markedly improved^68^.

In summary, existing studies have shown that EPDCs have achieved some results in improving cardiac function in rodents and hold early promise, but the administration of progenitor cells has not yet been translated into clinical practice. Their therapeutic effect is mainly mediated through secretory functions; however, the current cell retention rate is low. Therefore, it is necessary to improve the retention rate of therapeutic cells and enhance the release of key target factors^69^. In the treatment of myocardial injury, it is essential not only to focus on the delivery of exogenous cells but also to activate endogenous cells to ultimately replace lost cells and achieve repair. This includes stimulating endothelial cells to promote vascularization and inducing the proliferation of resident cardiomyocytes^70^. Tβ4, which promotes EPDCs inward migration and differentiation into endothelial and smooth muscle cells, is currently undergoing a multicenter phase I clinical trial for the treatment of cardiovascular diseases^71^, ^72^. However, there is still a long way to go before it can be widely applied in clinical practice.

## Challenges in the Application of the Epicardium for Heart Failure Treatment

Although research on the epicardium has opened up new avenues for the treatment of heart failure, its clinical translation remains in the early stages, with many aspects of the technology and underlying mechanisms still underdeveloped. To enable its widespread application in regenerative medicine, several key scientific and technical challenges must be systematically addressed (Table 2).

### Difficulty in Epicardial Cell Expansion and Differentiation *In Vitro*

Epicardial cells face significant challenges in terms of expansion and differentiation *in vitro*, mainly due to their limited quantity and lifespan. Although human epicardium and EPDCs hold great therapeutic potential, their limited availability hinders the advancement of related research. While EPDCs exhibit some proliferative ability, they often lose their epithelial phenotype after several passages, meaning that cells from different sources or passages may influence subsequent *in vitro* experimental outcomes^39^. Studies have shown that FGF-10 can promote the expansion of neonatal epicardial cells, but it does not significantly enhance cardiac regeneration^73^. Furthermore, when primary epicardial cells are isolated from E11.5 mouse hearts via tissue adhesion methods, they exhibit considerable genetic variability during passaging, particularly in the expression of EMT-related genes. The EpiSV40 cell line, transfected with SV40-LT, shows promising proliferation, migration, and differentiation potential *in vitro* and serves as a suitable model for epicardial research^74^.

### Post-transplantation Cell Survival and Functional Maintenance

Immune rejection and inflammatory responses are among the main challenges in cell transplantation therapy. Although Phase I clinical trials have demonstrated that autologous skeletal muscle-derived cardiomyocytes can improve cardiac function after myocardial infarction, they also increased the incidence of ventricular arrhythmias, likely due to the failure of transplanted cells to establish proper electromechanical coupling with host cardiomyocytes^75^-^78^. Currently, the optimal type and source of cardiac stem cells remain undefined, making it crucial to address how to enhance cell survival and maintain function post-transplantation while minimizing immune rejection—a central issue in cell-based therapies for heart failure.

### Issues of Biocompatibility and Degradability in Biomaterials

When using cellular or acellular biomaterials for cardiac repair, biocompatibility and mechanical properties are critical. Existing biomaterials often exhibit insufficient mechanical strength, making them incapable of providing adequate support to the damaged myocardium. For instance, many biomaterials have stiffness levels ranging from 10 to 20 Pa, whereas the stiffness of a healthy heart is approximately 50 kPa and that of a heart with congestive heart failure reaches 200-300 kPa. As a result, these materials are too soft during end-diastole to support the infarcted region effectively^77^. Moreover, biomaterials must be biodegradable to allow gradual degradation as myocardial function recovers and remodeling occurs, preventing long-term retention and potential adverse reactions. However, the gelation speed of many biomaterials is relatively slow, which could block blood flow and lead to tissue necrosis^79^.

Although cardiac patches have shown some progress, their clinical application is still limited. One key issue is the inability of the patches to achieve effective mechanical-electrical coupling with the host tissue, which can lead to arrhythmias. Therefore, developing biomaterials with improved mechanical strength, biocompatibility, and degradability remains a research priority.

### Safety and Efficacy of Gene Therapy

Gene therapy involves introducing exogenous genes into cells either *in vivo* or *in vitro* to treat or prevent disease. However, safety concerns remain a significant hurdle. The use of viral and non-viral vectors requires thorough safety assessments before clinical translation^80^. Although viral vectors such as adenoviruses and herpesviruses have been employed in gene therapy, they pose risks of tumorigenesis and immunogenicity, which need to be addressed^81^, ^82^. Additionally, while the CRISPR/Cas9 system holds great potential for correcting genetic mutations, it may trigger immune toxicity and off-target effects. Techniques such as Digenome-seq and CIRCLE-seq show promise but still suffer from limitations in detection accuracy and read length^83^. Therefore, minimizing off-target effects and immune toxicity will be critical for the safe application of gene-editing technologies in the future. Scientific and regulatory barriers to gene therapy development exist in many countries, limiting global market expansion. In addition, the limited capacity of existing good manufacturing practice (GMP) facilities and the long production cycles for clinical materials further increase time and costs^84^.

## Future Perspectives

In the study of epicardial therapy for heart failure, future innovations are likely to focus on the following key areas (Table 3):

### Enhancing the Expansion and Differentiation Capacity of Epicardium-Derived Cells

EPDCs have very limited ability to expand and differentiate *in vitro*. Future studies should aim to develop more efficient culture methods to overcome the loss of epithelial phenotype and multipotency during *in vitro* proliferation. Optimizing culture conditions, applying specific growth factors and cytokines, or regulating key signaling pathways such as Wnt/β-catenin and TGF-β through gene editing techniques may enhance EPDCs proliferation and differentiation, thereby improving their reparative capacity. Researchers may also explore combining EPDCs with other stem cell types to enhance their plasticity and regenerative potential. For example, culturing EPDCs in media containing SB431542, an ALK5 (TGF-β type I receptor) inhibitor, helps maintain their epithelial phenotype and allows for monolayer expansion**86**. In addition, co-culturing EPDCs with cardiac progenitor cells (CMPCs) isolated from human hearts has been shown to enhance EPDCs proliferation, as demonstrated by Ki67 analysis. When co-transplanted into infarcted mouse hearts, the combined cells exhibited a superior effect on cardiac remodeling through the secretion of matrix-modulating factors, showing synergistic benefits compared to single-cell-type therapies^85^.

### Development of Novel Biomaterials and Scaffolds

Current biomaterials and scaffolds for cardiac repair often lack sufficient mechanical strength and biocompatibility. Future efforts should focus on developing more elastic and biodegradable scaffolds that better mimic the mechanical properties of native cardiac tissue. Advances in 3D printing offer new possibilities for the precise design of biomaterials. This technology can be used to fabricate cardiac patches with optimized geometry, pore size, and diameter, thereby improving nutrient and oxygen transport, promoting cell proliferation, and enhancing cardiac function. Epicardium-derived ECM materials are also expected to play a key role in supporting cell proliferation, migration, and differentiation. Future research should explore how to integrate epicardium-derived ECM with biomaterials to enhance cardiac repair. For instance, the epicardium secretes hyaluronic acid (HA), which swells upon hydration and modulates epicardial cell EMT, migration, angiogenesis, and myocardial regeneration. After cardiac injury, integrin receptors on cardiomyocytes bind to fibronectin secreted by epicardial cells, enhancing regenerative signaling. Additionally, the marked upregulation of type XII collagen in the epicardium post-injury is closely linked to myocardial repair^87^.

### Further Optimization of Gene Therapy Technologies

As an innovative therapeutic strategy, gene therapy holds promise for breakthroughs in heart failure treatment. Advances in CRISPR/Cas9 technology have opened new possibilities for gene editing in EPDCs. Future studies should focus on optimizing the CRISPR/Cas9 system to minimize off-target effects while improving specificity and safety. Additionally, the development of novel non-viral vectors—such as nanoparticles and liposomes—will be key to enhancing delivery efficiency and targeting. Non-coding microRNAs (miRNAs), which can simultaneously target multiple mRNAs, have the potential to reprogram cardiomyocytes into a proliferative state. Adeno-associated viruses (AAVs) can selectively target cardiomyocytes, reducing the risk of ectopic gene expression in non-cardiac cells^88^. Integrating EPDCs with gene therapy may allow precise regulation of cardiac repair and facilitate functional recovery.

### Combining Immunomodulation with Cardiac Repair

Epicardial cells not only contribute to structural cardiac repair but also possess immunomodulatory capabilities that help reduce inflammation and adverse remodeling. Future research may focus on leveraging EPDCs to modulate immune responses and reduce rejection following heart or cell transplantation. Inducing EPDCs to secrete specific cytokines or exploiting their immunomodulatory functions could suppress pathological remodeling, improve cardiac function, and enhance clinical outcomes. For example, components of the epicardial Hippo signaling pathway, YAP and TAZ, can recruit regulatory T cells (Tregs) to the injured myocardium, suppressing post-infarction inflammation and limiting adverse remodeling^89^.

### Personalized Regenerative Strategies for Heart Failure

The future application of the epicardium in heart failure therapy is expected to move toward personalized medicine. With the advancement of precision medicine, treatment strategies can be tailored to individual patients based on specific disease conditions and the type of cardiac injury. For instance, in patients with heart failure with preserved ejection fraction (HFpEF), combining EPDCs with targeted gene therapy may help restore cardiac function. Developing personalized stem cell and gene therapy approaches based on individual immune status and genetic background could improve therapeutic efficacy and reduce side effects. For example, following cardiac injury, EPDCs can secrete Angpt4, which activates the Tie2-MAPK pathway in endocardial cells to promote regeneration. As a secreted protein, Angpt4 is easy to deliver and allows precise control over dosage and duration compared to intracellular proteins^90^. Nanoparticles, capable of carrying multiple targeted molecules, offer flexibility in drug delivery and may pave the way for personalized medicine^91^. Additionally, systems have been developed to maintain the epithelial phenotype of adult human epicardial cells *in vitro*, and methods exist to induce pluripotent stem cells to differentiate into epicardial-like cells via Wnt signaling modulation. These patient-derived cell types offer a feasible path toward individualized cardiac therapy^92^.

## Summary

The epicardium and EPDCs, with their intrinsic potential to differentiate into multiple cardiac lineages—including cardiomyocytes—have emerged as a promising cell source for cardiac regenerative therapy. Their key roles in subepicardial matrix remodeling and paracrine signaling further enhance their therapeutic appeal. Increasing evidence supports the involvement of the epicardium and EPDCs in heart failure treatment. However, challenges remain in maintaining their phenotypic stability and multipotency during *in vitro* expansion, as well as ensuring the safety, efficacy, and personalization of gene therapies. Future research should focus on harnessing the multipotent capacity of EPDCs by identifying optimal stimulatory factors, integrating gene editing technologies, and combining them with advanced biomaterials to enhance their regenerative potential and promote differentiation into functional cardiomyocytes. Personalized treatment strategies tailored to individual patient profiles may further improve therapeutic outcomes and provide strong support for the advancement of heart failure therapies.

## Figures and Tables

**Table 1:** Effects of Different Influencing Factors on EPDCs

Effects	Influencing factors	References
Differentiation potential under different conditions	Ø Atrial natriuretic peptide can promote its differentiation into adipocytes Ø Angiotensin II promotes differentiation into myofibroblasts Ø Overexpression of Cdx1 promotes differentiation into coronary artery SMCs and endothelial cells Ø TGF-β receptor promotes differentiation into SMCs Ø FGF1/2/7 can induce SMCs differentiation	5, 35, 36
Influence EMT in epicardial cells	Ø Overexpression of Cdx1 c Ø TGF-β receptor stimulation Ø PDGF receptors and FGF1/2/7 Ø ALK5 kinase inhibitor SB431542 inhibits EMT in adult EPDCs, but has little effect on fetuses	5, 36, 38
Influence proliferation and migration	Ø Tβ4 has been shown to enhance the migratory capacity of adult EPDCs Ø Inducible LT expression system regulated by doxycycline enables high proliferation capacity	37, 39

Abbreviation: SMCs: smooth muscle cells; TGF-β: transforming factor β; PDGF: platelet-derived growth factor; FGF: fibroblast growth factor; LT: large T-antigen.

**Table 2:** Brief Table of Key Challenges in the Application of the Epicardium for Heart Failure Treatment

Challenges	Specific Problems	References
Difficulty in epicardial cell expansion and differentiation *in vitro*	Ø Epicardial cells have limited quantity and lifespan, hindering *in vitro* expansion Ø Loss of epithelial phenotype after passaging Ø Significant variability among cells from different sources or passages Ø Existing proliferation factors (e.g., FGF-10) have limited regenerative effects; primary cells exhibit genetic variability during culture; available cell lines (e.g., EpiSV40) require further validation for clinical use	39, 73, 74
Poor survival and functional maintenance post-cell transplantation	Ø Transplanted cells are susceptible to immune rejection and inflammatory damage Ø Challenges exist in achieving effective electromechanical coupling between transplanted and host cardiomyocytes, increasing risk of arrhythmias Ø Optimal type and source of cardiac stem cells are still undefined Ø Ensuring enhanced cell survival and sustained functionality post-transplantation remains a critical challenge	75-78
Issues of biocompatibility and degradability in biomaterials	Ø Current biomaterials lack sufficient mechanical strength to support damaged myocardium (healthy myocardium stiffness ~50 kPa, heart failure myocardium ~200-300 kPa Ø Current biomaterials are only ~10-20 Pa) Ø Slow gelation rate of hydrogels may lead to blood flow obstruction and tissue necrosis Ø Biomaterials need appropriate biodegradability matched with myocardial repair process to prevent long-term retention and adverse reactions	79, 80
Safety and efficacy concerns in gene therapy	Ø Viral or non-viral vectors in gene therapy carry risks of tumorigenicity and immunogenicity CRISPR/Cas9 gene-editing technology faces immune toxicity and off-target effects Ø Current detection techniques (e.g., Digenome-seq, CIRCLE-seq) have limited precision, highlighting the need for reducing off-target effects and immune toxicity	80-83

**Table 3:** A brief summary of future directions for epicardial therapy in heart failure

Future Directions	Potential Strategies and Innovations	References
Enhancing Expansion and Differentiation of EPDCs	Optimize culture conditions and signaling pathways (Wnt/β-catenin, TGF-β) Employ SB431542 to maintain epithelial phenotype Co-culture EPDCs with cardiac progenitor cells (CMPCs) to enhance proliferation and regenerative capabilities	85, 86
Development of Novel Biomaterials and Scaffolds	Create elastic, biodegradable scaffolds mimicking cardiac mechanics Apply advanced 3D printing techniques for cardiac patch fabrication Integrate epicardium-derived ECM components (e.g., hyaluronic acid, fibronectin, type XII collagen) into biomaterial designs	87
Optimization of Gene Therapy Technologies	Refine CRISPR/Cas9 methods to reduce off-target effects Develop non-viral delivery systems (nanoparticles, liposomes) Utilize microRNAs and AAV vectors for targeted gene delivery and cardiac reprogramming	88
Combining Immunomodulation with Cardiac Repair	Exploit immunomodulatory effects of EPDCs-secreted cytokines Utilize Hippo pathway (YAP/TAZ) to recruit regulatory T cells, mitigating inflammation and adverse remodeling	89
Personalized Regenerative Strategies for Heart Failure	Tailor EPDCs and gene therapy approaches to individual genetic profiles and immune status Deliver Angpt4 protein precisely via nanoparticles for controlled regenerative responses Utilize patient-derived epicardial-like cells from induced pluripotent stem cells	90-92

## References

[1] Khan MS, Shahid I, Bennis A, Rakisheva A, Metra M, Butler J (2024). Global epidemiology of heart failure. Nat Rev Cardiol.

[2] Schwinger RHG (2021). Pathophysiology of heart failure. Cardiovasc Diagn Ther.

[3] Rosenbaum AN, Agre KE, Pereira NL (2020). Genetics of dilated cardiomyopathy: practical implications for heart failure management. Nat Rev Cardiol.

[4] Vieira JM, Riley PR (2011). Epicardium-derived cells: a new source of regenerative capacity. Heart.

[5] Cao Y, Duca S, Cao J (2020). Epicardium in Heart Development. Cold Spring Harb Perspect Biol.

[6] Quijada P, Trembley MA, Small EM (2020). The Role of the Epicardium During Heart Development and Repair. Circ Res.

[7] Suffee N, Moore-Morris T, Jagla B, Mougenot N, Dilanian G, Berthet M (2020). Reactivation of the Epicardium at the Origin of Myocardial Fibro-Fatty Infiltration During the Atrial Cardiomyopathy. Circ Res.

[8] Ruiz-Villalba A, Simon AM, Pogontke C, Castillo MI, Abizanda G, Pelacho B (2015). Interacting resident epicardium-derived fibroblasts and recruited bone marrow cells form myocardial infarction scar. J Am Coll Cardiol.

[9] Smits AM, Dronkers E, Goumans MJ (2018). The epicardium as a source of multipotent adult cardiac progenitor cells: Their origin, role and fate. Pharmacol Res.

[10] Männer J, Perez-Pomares J, Macias D, Munoz-Chapuli R (2001). The origin, formation and developmental significance of the epicardium: a review. Cells Tissues Organs.

[11] Balbi C, Smart N (2023). Epicardioids: a novel tool for cardiac regeneration research?. Cardiovasc Res.

[12] Dronkers E, Wauters MMM, Goumans MJ, Smits AM (2020). Epicardial TGFbeta and BMP Signaling in Cardiac Regeneration: What Lesson Can We Learn from the Developing Heart?. Biomolecules.

[13] Smart N, Riley PR (2012). The epicardium as a candidate for heart regeneration. Future Cardiol.

[14] Lavine KJ, White AC, Park C, Smith CS, Choi K, Long F (2006). Fibroblast growth factor signals regulate a wave of Hedgehog activation that is essential for coronary vascular development. Genes Dev.

[15] Lavine KJ, Long F, Choi K, Smith C, Ornitz DM (2008). Hedgehog signaling to distinct cell types differentially regulates coronary artery and vein development. Development.

[16] Huang GN, Thatcher JE, McAnally J, Kong Y, Qi X, Tan W (2012). C/EBP transcription factors mediate epicardial activation during heart development and injury. Science.

[17] Peterson EA, Sun J, Chen X, Wang J (2024). Neutrophils facilitate the epicardial regenerative response after zebrafish heart injury. Dev Biol.

[18] Bargehr J, Ong LP, Colzani M, Davaapil H, Hofsteen P, Bhandari S (2019). Epicardial cells derived from human embryonic stem cells augment cardiomyocyte-driven heart regeneration. Nat Biotechnol.

[19] von Gise A, Pu WT (2012). Endocardial and epicardial epithelial to mesenchymal transitions in heart development and disease. Circ Res.

[20] Smits AM, Riley PR (2014). Epicardium-Derived Heart Repair. J Dev Biol.

[21] Lamouille S, Xu J, Derynck R (2014). Molecular mechanisms of epithelial-mesenchymal transition. Nat Rev Mol Cell Biol.

[22] Eroglu E, Yen CYT, Tsoi YL, Witman N, Elewa A, Joven Araus A (2022). Epicardium-derived cells organize through tight junctions to replenish cardiac muscle in salamanders. Nat Cell Biol.

[23] Sanchez-Fernandez C, Rodriguez-Outeirino L, Matias-Valiente L, Ramirez de Acuna F, Franco D, Aranega AE (2023). Understanding Epicardial Cell Heterogeneity during Cardiogenesis and Heart Regeneration. J Cardiovasc Dev Dis.

[24] Cao J, Poss KD (2018). The epicardium as a hub for heart regeneration. Nat Rev Cardiol.

[25] Stevens SM, von Gise A, VanDusen N, Zhou B, Pu WT (2016). Epicardium is required for cardiac seeding by yolk sac macrophages, precursors of resident macrophages of the adult heart. Dev Biol.

[26] Zhou B, Honor LB, He H, Ma Q, Oh JH, Butterfield C (2011). Adult mouse epicardium modulates myocardial injury by secreting paracrine factors. J Clin Invest.

[27] Zhou B, Honor LB, Ma Q, Oh JH, Lin RZ, Melero-Martin JM (2012). Thymosin beta 4 treatment after myocardial infarction does not reprogram epicardial cells into cardiomyocytes. J Mol Cell Cardiol.

[28] Smart N, Risebro CA, Clark JE, Ehler E, Miquerol L, Rossdeutsch A (2010). Thymosin beta4 facilitates epicardial neovascularization of the injured adult heart. Ann N Y Acad Sci.

[29] van Tuyn J, Atsma DE, Winter EM, van der Velde-van Dijke I, Pijnappels DA, Bax NA (2007). Epicardial cells of human adults can undergo an epithelial-to-mesenchymal transition and obtain characteristics of smooth muscle cells *in vitro*. Stem Cells.

[30] Lavine KJ, Yu K, White AC, Zhang X, Smith C, Partanen J (2005). Endocardial and epicardial derived FGF signals regulate myocardial proliferation and differentiation *in vivo*. Dev Cell.

[31] Merki E, Zamora M, Raya A, Kawakami Y, Wang J, Zhang X (2005). Epicardial retinoid X receptor α is required for myocardial growth and coronary artery formation. Proceedings of the National Academy of Sciences.

[32] Chen T, Chang TC, Kang JO, Choudhary B, Makita T, Tran CM (2002). Epicardial induction of fetal cardiomyocyte proliferation via a retinoic acid-inducible trophic factor. Dev Biol.

[33] Lepilina A, Coon AN, Kikuchi K, Holdway JE, Roberts RW, Burns CG (2006). A dynamic epicardial injury response supports progenitor cell activity during zebrafish heart regeneration. Cell.

[34] Yasmeen S, Liao X, Khan FU, Ihsan AU, Li X, Li C (2019). A novel approach to devise the therapy for ventricular fibrillation by epicardial delivery of lidocaine using active hydraulic ventricular attaching support system: An experimental study in rats. J Biomed Mater Res B Appl Biomater.

[35] Van Wagoner DR (2020). Paracrine signals modulate atrial epicardial progenitor cells and development of subepicardial adiposity and fibrosis implications for atrial fibrillation. Lippincott Williams & Wilkins Hagerstown, MD.

[36] Chu M, Wang L, Wang H, Shen T, Yang Y, Sun Y (2014). A novel role of CDX1 in embryonic epicardial development. PLoS One.

[37] Smart N, Riley PR (2009). Derivation of epicardium-derived progenitor cells (EPDCs) from adult epicardium. Curr Protoc Stem Cell Biol.

[38] Dronkers E, Moerkamp AT, van Herwaarden T, Goumans MJ, Smits AM (2018). The Isolation and Culture of Primary Epicardial Cells Derived from Human Adult and Fetal Heart Specimens. J Vis Exp.

[39] Ge Y, Smits AM, Liu J, Zhang J, van Brakel TJ, Goumans M (2021). Generation, Characterization, and Application of Inducible Proliferative Adult Human Epicardium-Derived Cells. Cells.

[40] Zhu D, Liu S, Huang K, Li J, Mei X, Li Z (2023). Intrapericardial long non-coding RNA-Tcf21 antisense RNA inducing demethylation administration promotes cardiac repair. Eur Heart J.

[41] Blom JN, Wang X, Lu X, Kim MY, Wang G, Feng Q (2022). Inhibition of intraflagellar transport protein-88 promotes epithelial-to-mesenchymal transition and reduces cardiac remodeling post-myocardial infarction. European Journal of Pharmacology.

[42] Ma X, Zhao J, Feng Y (2024). Epicardial SMARCA4 deletion exacerbates cardiac injury in myocardial infarction and is related to the inhibition of epicardial epithelial-mesenchymal transition. Journal of Molecular and Cellular Cardiology.

[43] Wasserman AH, Huang AR, Lewis-Israeli YR, Dooley MD, Mitchell AL, Venkatesan M (2022). Oxytocin promotes epicardial cell activation and heart regeneration after cardiac injury. Frontiers in Cell and Developmental Biology.

[44] Li J, Li S-H, Wu J, Weisel RD, Yao A, Stanford WL (2018). Young bone marrow Sca-1 cells rejuvenate the aged heart by promoting epithelial-to-mesenchymal transition. Theranostics.

[45] Xu Y, Zhang X, Fu Z, Dong Y, Yu Y, Liu Y (2024). Intrapericardial Administration of Human Pericardial Fluid Cells Improves Cardiac Functions in Rats with Heart Failure. Stem Cells and Development.

[46] Xu Y, Chen Y, Tan JJ, Ooi JP, Guo Z (2025). Intrapericardial Administration to Achieve Localized and Targeted Treatment for Cardiac Disease. J Cardiovasc Transl Res.

[47] Wang J, Karra R, Dickson AL, Poss KD (2013). Fibronectin is deposited by injury-activated epicardial cells and is necessary for zebrafish heart regeneration. Dev Biol.

[48] Kuhn B, del Monte F, Hajjar RJ, Chang YS, Lebeche D, Arab S (2007). Periostin induces proliferation of differentiated cardiomyocytes and promotes cardiac repair. Nat Med.

[49] Becker M Development of a bioactive epicardial composite patch for support of myocardial regeneration. 2020.

[50] Park J, Choi S, Janardhan AH, Lee S-Y, Raut S, Soares J (2016). Electromechanical cardioplasty using a wrapped elasto-conductive epicardial mesh. Science translational medicine.

[51] Li M, Wu H, Yuan Y, Hu B, Gu N (2022). Recent fabrications and applications of cardiac patch in myocardial infarction treatment. View.

[52] Wei K, Serpooshan V, Hurtado C, Diez-Cunado M, Zhao M, Maruyama S (2015). Epicardial FSTL1 reconstitution regenerates the adult mammalian heart. Nature.

[53] Murphy SV, Atala A (2014). 3D bioprinting of tissues and organs. Nat Biotechnol.

[54] Theus AS, Ning L, Hwang B, Gil C, Chen S, Wombwell A (2020). Bioprintability: Physiomechanical and Biological Requirements of Materials for 3D Bioprinting Processes. Polymers (Basel).

[55] Chong JJ, Yang X, Don CW, Minami E, Liu YW, Weyers JJ (2014). Human embryonic-stem-cell-derived cardiomyocytes regenerate non-human primate hearts. Nature.

[56] Zhang Y, Mu W, Zhang Y, He X, Wang Y, Ma H (2022). Recent Advances in Cardiac Patches: Materials, Preparations, and Properties. ACS Biomater Sci Eng.

[57] Nakamura K, Henry TD, Traverse JH, Latter DA, Mokadam NA, Answini GA (2024). Angiogenic Gene Therapy for Refractory Angina: Results of the EXACT Phase 2 Trial. Circ Cardiovasc Interv.

[58] Maslov M, Foianini S, Lovich M (2017). Delivery of drugs, growth factors, genes and stem cells via intrapericardial, epicardial and intramyocardial routes for sustained local targeted therapy of myocardial disease. Expert Opin Drug Deliv.

[59] Hosseinkhani H, Domb AJ, Sharifzadeh G, Nahum V (2023). Gene Therapy for Regenerative Medicine. Pharmaceutics.

[60] Benson JM, Wang G, Hutt JA, Wu G, Kaminsky SM, Cram S (2023). Preclinical safety and biodistribution assessment of Ad-KCNH2-G628S administered via atrial painting in New Zealand white rabbits. Basic Clin Pharmacol Toxicol.

[61] Kikuchi K, McDonald AD, Sasano T, Donahue JK (2005). Targeted modification of atrial electrophysiology by homogeneous transmural atrial gene transfer. Circulation.

[62] Liu Z, Hutt JA, Rajeshkumar B, Azuma Y, Duan KL, Donahue JK (2017). Preclinical efficacy and safety of KCNH2-G628S gene therapy for postoperative atrial fibrillation. J Thorac Cardiovasc Surg.

[63] Mo W, Donahue JK (2024). Gene therapy for atrial fibrillation. J Mol Cell Cardiol.

[64] Mendez KL, Varela CE, Bonnemain J, Deng J, Yuk H, Ayers B (2024). SmartSleeve: A sutureless, soft robotic epicardial device that enables switchable on-off drug delivery in response to epicardial ECG sensing. Device.

[65] Hsu PD, Lander ES, Zhang F (2014). Development and applications of CRISPR-Cas9 for genome engineering. Cell.

[66] Doudna JA, Charpentier E (2014). The new frontier of genome engineering with CRISPR-Cas9. Science.

[67] Park H, Kim D, Cho B, Byun J, Kim YS, Ahn Y (2022). *In vivo* therapeutic genome editing via CRISPR/Cas9 magnetoplexes for myocardial infarction. Biomaterials.

[68] Wang YL, Yu SN, Shen HR, Wang HJ, Wu XP, Wang QL (2021). Thymosin beta4 released from functionalized self-assembling peptide activates epicardium and enhances repair of infarcted myocardium. Theranostics.

[69] Alonaizan R, Carr C (2022). Cardiac regeneration following myocardial infarction: the need for regeneration and a review of cardiac stromal cell populations used for transplantation. Biochemical Society Transactions.

[70] Streef TJ, Smits AM (2021). Epicardial contribution to the developing and injured heart: exploring the cellular composition of the epicardium. Frontiers in Cardiovascular Medicine.

[71] Krainock M, Toubat O, Danopoulos S, Beckham A, Warburton D, Kim R (2016). Epicardial epithelial-to-mesenchymal transition in heart development and disease. Journal of clinical medicine.

[72] Smart N, Risebro CA, Melville AA, Moses K, Schwartz RJ, Chien KR (2007). Thymosin β-4 is essential for coronary vessel development and promotes neovascularization via adult epicardium. Annals of the New York Academy of Sciences.

[73] Rubin N, Darehzereshki A, Bellusci S, Kaartinen V, Ling Lien C (2013). FGF10 Signaling Enhances Epicardial Cell Expansion during Neonatal Mouse Heart Repair. J Cardiovasc Dis Diagn.

[74] Jiang H, Song S, Li J, Yin Q, Hu S, Nie Y (2021). Establishment and characterization of an immortalized epicardial cell line. J Cell Mol Med.

[75] Léobon B, Garcin I, Menasché P, Vilquin J-T, Audinat E, Charpak S (2003). Myoblasts transplanted into rat infarcted myocardium are functionally isolated from their host. Proceedings of the National Academy of Sciences.

[76] Hagege AA, Marolleau JP, Vilquin JT, Alheritiere A, Peyrard S, Duboc D (2006). Skeletal myoblast transplantation in ischemic heart failure: long-term follow-up of the first phase I cohort of patients. Circulation.

[77] Fernandes S, Amirault JC, Lande G, Nguyen JM, Forest V, Bignolais O (2006). Autologous myoblast transplantation after myocardial infarction increases the inducibility of ventricular arrhythmias. Cardiovasc Res.

[78] Menasche P, Alfieri O, Janssens S, McKenna W, Reichenspurner H, Trinquart L (2008). The Myoblast Autologous Grafting in Ischemic Cardiomyopathy (MAGIC) trial: first randomized placebo-controlled study of myoblast transplantation. Circulation.

[79] Taylor DA, Hruban R, Rodriguez ER, Goldschmidt-Clermont PJ (2002). Cardiac chimerism as a mechanism for self-repair: does it happen and if so to what degree?. Circulation.

[80] Ghosh S, Brown AM, Jenkins C, Campbell K (2020). Viral Vector Systems for Gene Therapy: A Comprehensive Literature Review of Progress and Biosafety Challenges. Appl Biosaf.

[81] Fears R, Ter Meulen V (2018). Assessing Security Implications of Genome Editing: Emerging Points From an International Workshop. Front Bioeng Biotechnol.

[82] Almarza D, Bussadori G, Navarro M, Mavilio F, Larcher F, Murillas R (2011). Risk assessment in skin gene therapy: viral-cellular fusion transcripts generated by proviral transcriptional read-through in keratinocytes transduced with self-inactivating lentiviral vectors. Gene Ther.

[83] Ihry RJ, Worringer KA, Salick MR, Frias E, Ho D, Theriault K (2018). p53 inhibits CRISPR-Cas9 engineering in human pluripotent stem cells. Nat Med.

[84] Drago D, Foss-Campbell B, Wonnacott K, Barrett D, Ndu A (2021). Global regulatory progress in delivering on the promise of gene therapies for unmet medical needs. Molecular Therapy Methods & Clinical Development.

[85] Winter EM, van Oorschot AA, Hogers B, van der Graaf LM, Doevendans PA, Poelmann RE (2009). A new direction for cardiac regeneration therapy: application of synergistically acting epicardium-derived cells and cardiomyocyte progenitor cells. Circ Heart Fail.

[86] Moerkamp AT, Lodder K, van Herwaarden T, Dronkers E, Dingenouts CK, Tengstrom FC (2016). Human fetal and adult epicardial-derived cells: a novel model to study their activation. Stem Cell Res Ther.

[87] Saifi O, Ghandour B, Jaalouk D, Refaat M, Mahfouz R (2019). Myocardial regeneration: role of epicardium and implicated genes. Mol Biol Rep.

[88] Van Linthout S, Stellos K, Giacca M, Bertero E, Cannata A, Carrier L (2025). State of the art and perspectives of gene therapy in heart failure. A scientific statement of the Heart Failure Association of the ESC, the ESC Council on Cardiovascular Genomics and the ESC Working Group on Myocardial & Pericardial Diseases. Eur J Heart Fail.

[89] Ramjee V, Li D, Manderfield LJ, Liu F, Engleka KA, Aghajanian H (2017). Epicardial YAP/TAZ orchestrate an immunosuppressive response following myocardial infarction. J Clin Invest.

[90] Wu Z, Shi Y, Cui Y, Xing X, Zhang L, Liu D (2023). Single-cell analysis reveals an Angpt4-initiated EPDC-EC-CM cellular coordination cascade during heart regeneration. Protein Cell.

[91] Tariq U, Gupta M, Pathak S, Patil R, Dohare A, Misra SK (2022). Role of Biomaterials in Cardiac Repair and Regeneration: Therapeutic Intervention for Myocardial Infarction. ACS Biomater Sci Eng.

[92] Foglio E, D'Avorio E, Nieri R, Russo MA, Limana F (2024). Epicardial EMT and cardiac repair: an update. Stem Cell Res Ther.

